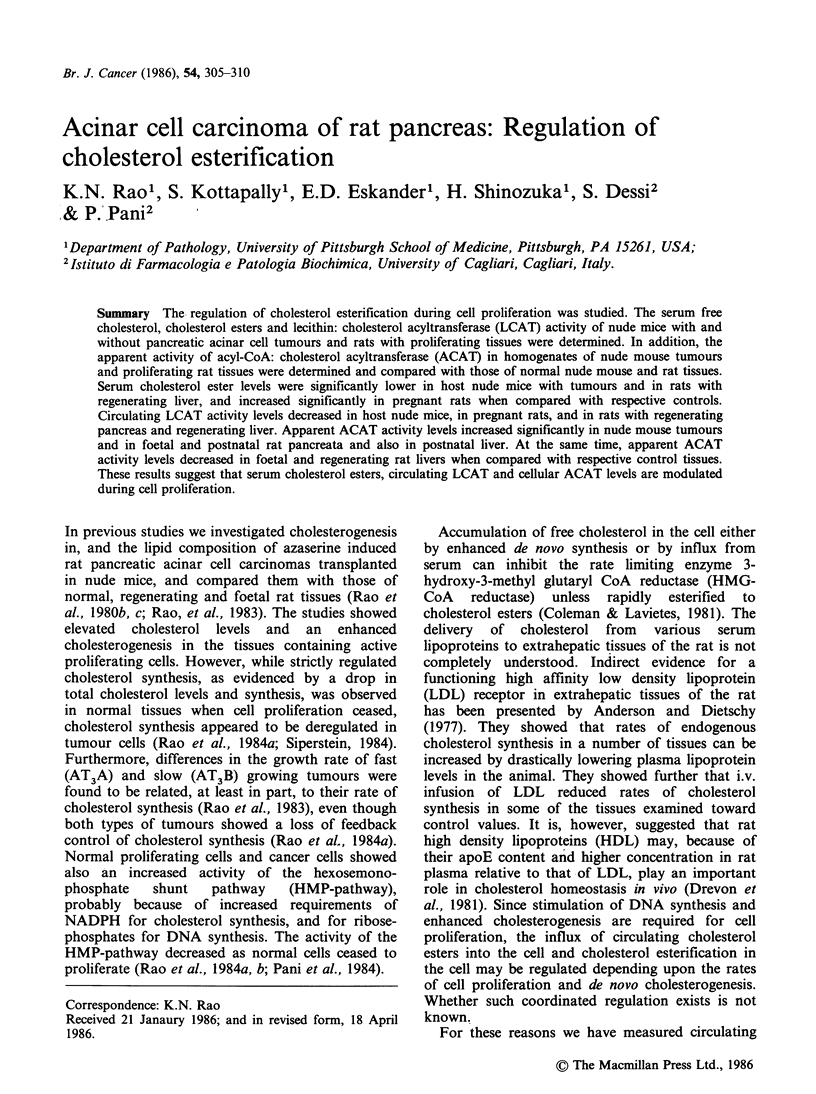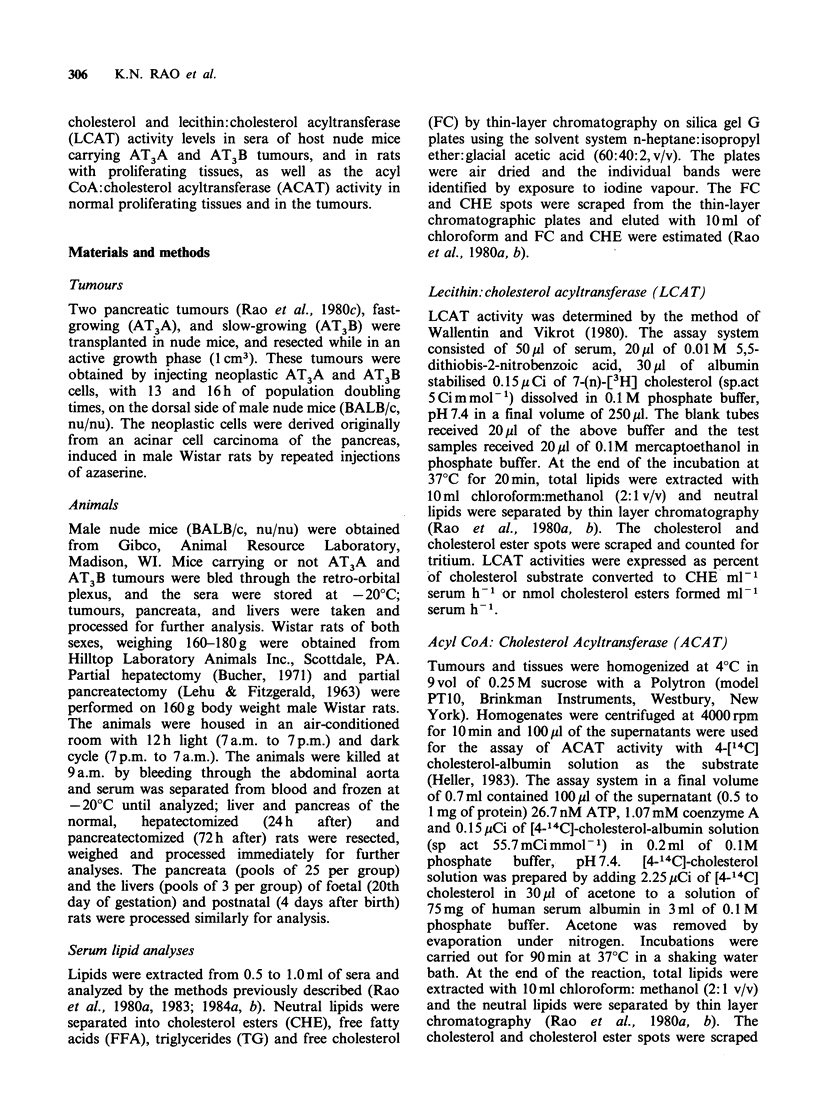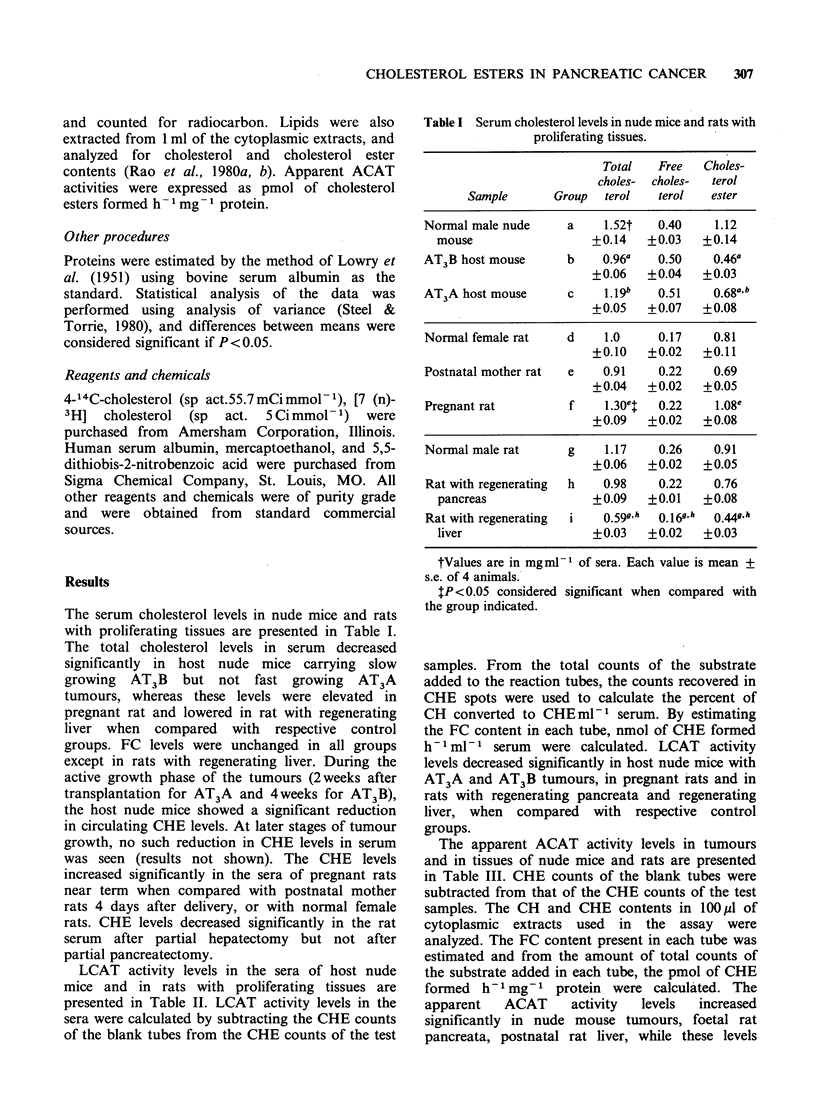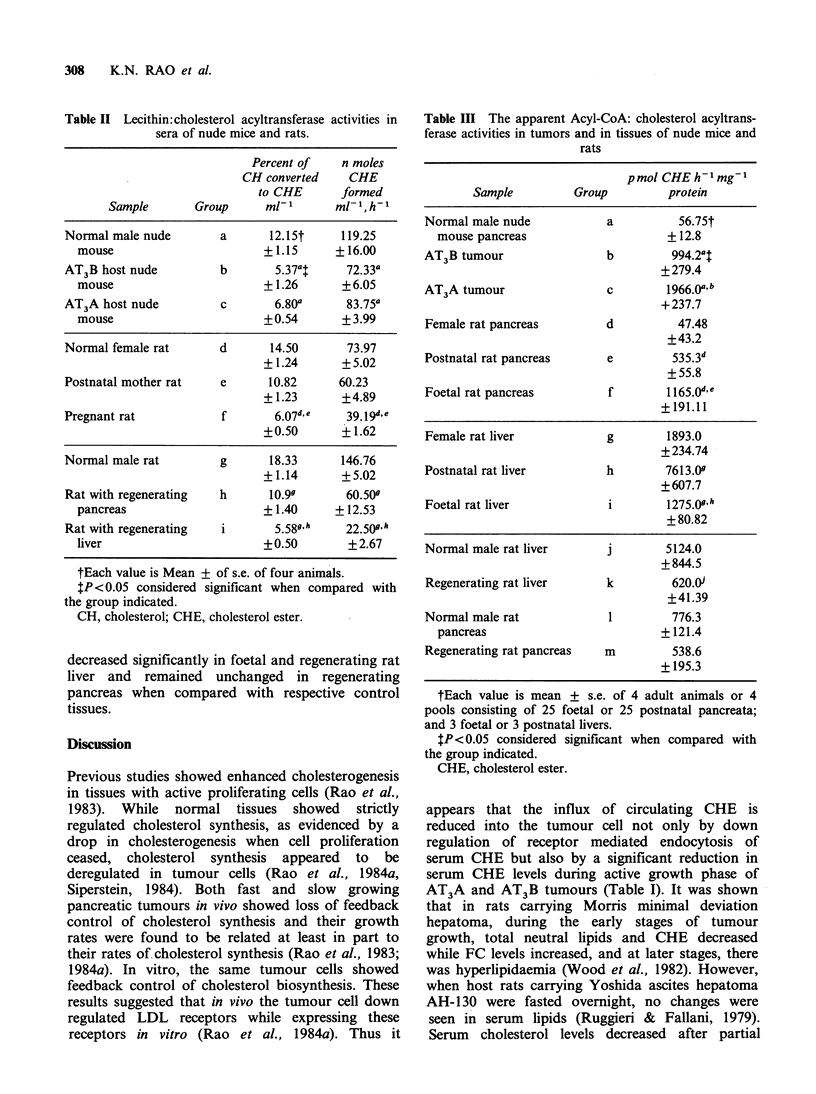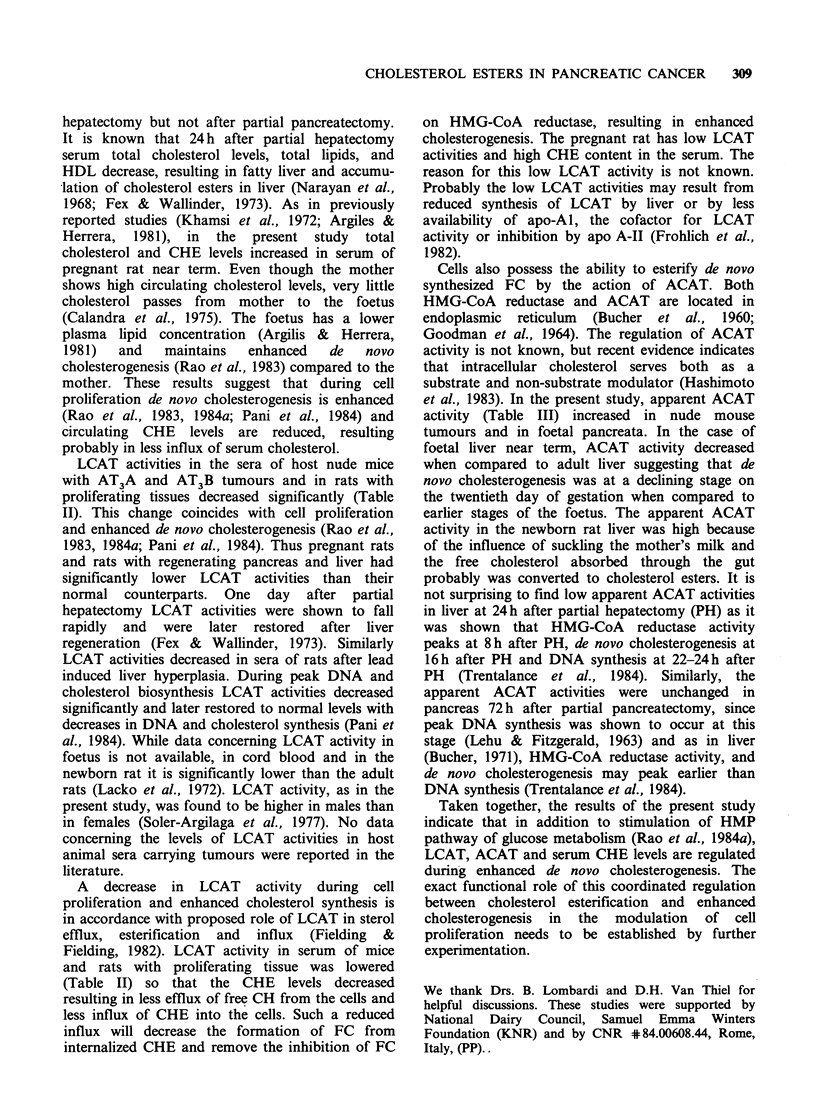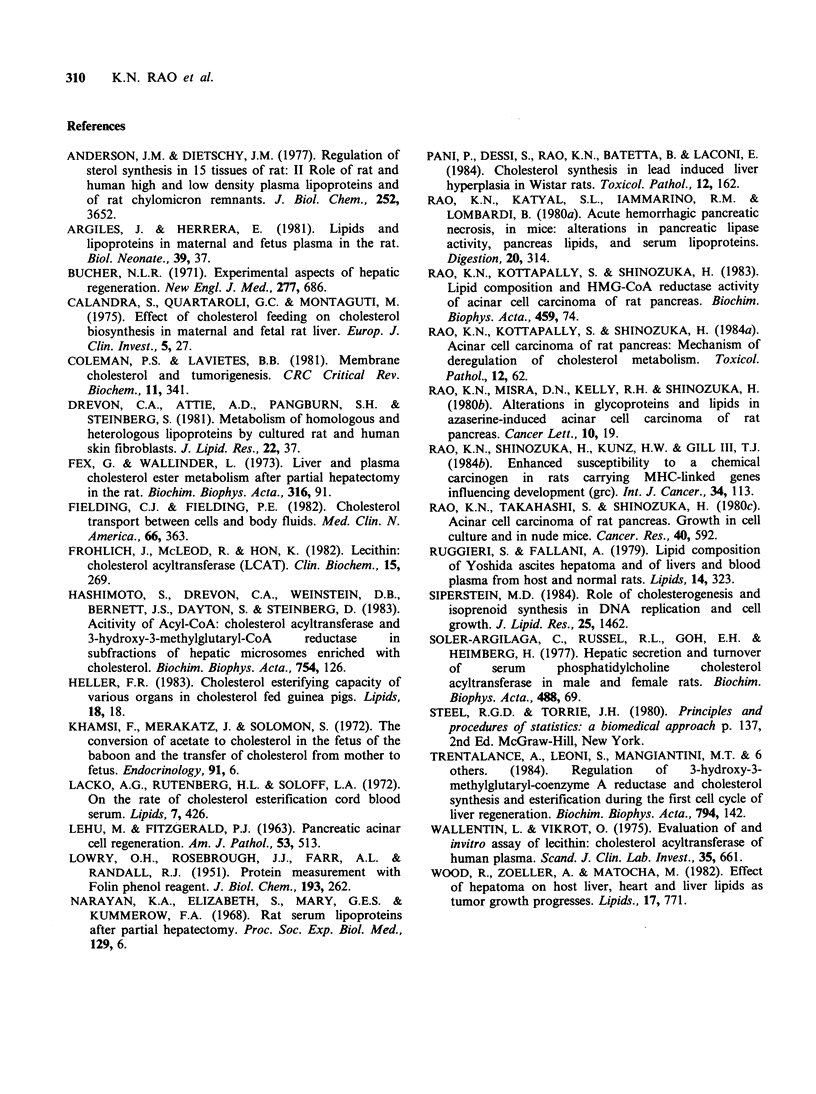# Acinar cell carcinoma of rat pancreas: regulation of cholesterol esterification.

**DOI:** 10.1038/bjc.1986.177

**Published:** 1986-08

**Authors:** K. N. Rao, S. Kottapally, E. D. Eskander, H. Shinozuka, S. Dessi, P. Pani

## Abstract

The regulation of cholesterol esterification during cell proliferation was studied. The serum free cholesterol, cholesterol esters and lecithin: cholesterol acyltransferase (LCAT) activity of nude mice with and without pancreatic acinar cell tumours and rats with proliferating tissues were determined. In addition, the apparent activity of acyl-CoA: cholesterol acyltransferase (ACAT) in homogenates of nude mouse tumours and proliferating rat tissues were determined and compared with those of normal nude mouse and rat tissues. Serum cholesterol ester levels were significantly lower in host nude mice with tumours and in rats with regenerating liver, and increased significantly in pregnant rats when compared with respective controls. Circulating LCAT activity levels decreased in host nude mice, in pregnant rats, and in rats with regenerating pancreas and regenerating liver. Apparent ACAT activity levels increased significantly in nude mouse tumours and in foetal and postnatal rat pancreata and also in postnatal liver. At the same time, apparent ACAT activity levels decreased in foetal and regenerating rat livers when compared with respective control tissues. These results suggest that serum cholesterol esters, circulating LCAT and cellular ACAT levels are modulated during cell proliferation.


*Br. J. Cancer* (1986), **54**, 305–310

### Acinar cell carcinoma of rat pancreas: Regulation of cholesterol esterification

K.N. Rao[1], S. Kottapally[1], E.D. Eskander[1], H. Shinozuka[1], S. Dessi[2] & P. Pani[2]

[1]*Department of Pathology, University of Pittsburgh School of Medicine, Pittsburgh, PA 15261, USA;*
[2]*Istituto di Farmacologia e Patologia Biochimica, University of Cagliari, Cagliari, Italy.*

**Summary** The regulation of cholesterol esterification during cell proliferation was studied. The serum free cholesterol, cholesterol esters and lecithin: cholesterol acyltransferase (LCAT) activity of nude mice with and without pancreatic acinar cell tumours and rats with proliferating tissues were determined. In addition, the apparent activity of acyl-CoA: cholesterol acyltransferase (ACAT) in homogenates of nude mouse tumours and proliferating rat tissues were determined and compared with those of normal nude mouse and rat tissues. Serum cholesterol ester levels were significantly lower in host nude mice with tumours and in rats with regenerating liver, and increased significantly in pregnant rats when compared with respective controls. Circulating LCAT activity levels decreased in host nude mice, in pregnant rats, and in rats with regenerating pancreas and regenerating liver. Apparent ACAT activity levels increased significantly in nude mouse tumours and in foetal and postnatal rat pancreata and also in postnatal liver. At the same time, apparent ACAT activity levels decreased in foetal and regenerating rat livers when compared with respective control tissues. These results suggest that serum cholesterol esters, circulating LCAT and cellular ACAT levels are modulated during cell proliferation.

In previous studies we investigated cholesterogenesis in, and the lipid composition of azaserine induced rat pancreatic acinar cell carcinomas transplanted in nude mice, and compared them with those of normal, regenerating and foetal rat tissues (Rao *et al.*, 1980*b*, *c*; Rao, *et al.*, 1983). The studies showed elevated cholesterol levels and an enhanced cholesterogenesis in the tissues containing active proliferating cells. However, while strictly regulated cholesterol synthesis, as evidenced by a drop in total cholesterol levels and synthesis, was observed in normal tissues when cell proliferation ceased, cholesterol synthesis appeared to be deregulated in tumour cells (Rao *et al.*, 1984*a*; Siperstein, 1984). Furthermore, differences in the growth rate of fast ($AT_3A$) and slow ($AT_3B$) growing tumours were found to be related, at least in part, to their rate of cholesterol synthesis (Rao *et al.*, 1983), even though both types of tumours showed a loss of feedback control of cholesterol synthesis (Rao *et al.*, 1984*a*). Normal proliferating cells and cancer cells showed also an increased activity of the hexosemonophosphate shunt pathway (HMP-pathway), probably because of increased requirements of NADPH for cholesterol synthesis, and for ribose-phosphates for DNA synthesis. The activity of the HMP-pathway decreased as normal cells ceased to proliferate (Rao *et al.*, 1984*a*, *b*; Pani *et al.*, 1984).

Correspondence: K.N. Rao
Received 21 Janaury 1986; and in revised form, 18 April 1986.

Accumulation of free cholesterol in the cell either by enhanced *de novo* synthesis or by influx from serum can inhibit the rate limiting enzyme 3-hydroxy-3-methyl glutaryl CoA reductase (HMG-CoA reductase) unless rapidly esterified to cholesterol esters (Coleman & Lavietes, 1981). The delivery of cholesterol from various serum lipoproteins to extrahepatic tissues of the rat is not completely understood. Indirect evidence for a functioning high affinity low density lipoprotein (LDL) receptor in extrahepatic tissues of the rat has been presented by Anderson and Dietschy (1977). They showed that rates of endogenous cholesterol synthesis in a number of tissues can be increased by drastically lowering plasma lipoprotein levels in the animal. They showed further that i.v. infusion of LDL reduced rates of cholesterol synthesis in some of the tissues examined toward control values. It is, however, suggested that rat high density lipoproteins (HDL) may, because of their apoE content and higher concentration in rat plasma relative to that of LDL, play an important role in cholesterol homeostasis *in vivo* (Drevon *et al.*, 1981). Since stimulation of DNA synthesis and enhanced cholesterogenesis are required for cell proliferation, the influx of circulating cholesterol esters into the cell and cholesterol esterification in the cell may be regulated depending upon the rates of cell proliferation and *de novo* cholesterogenesis. Whether such coordinated regulation exists is not known.

For these reasons we have measured circulating

© The Macmillan Press Ltd., 1986

cholesterol and lecithin:cholesterol acyltransferase (LCAT) activity levels in sera of host nude mice carrying $AT_3A$ and $AT_3B$ tumours, and in rats with proliferating tissues, as well as the acyl CoA:cholesterol acyltransferase (ACAT) activity in normal proliferating tissues and in the tumours.

### Materials and methods

### *Tumours*

Two pancreatic tumours (Rao *et al.*, 1980*c*), fast-growing ($AT_3A$), and slow-growing ($AT_3B$) were transplanted in nude mice, and resected while in an active growth phase (1 $cm^3$). These tumours were obtained by injecting neoplastic $AT_3A$ and $AT_3B$ cells, with 13 and 16 h of population doubling times, on the dorsal side of male nude mice (BALB/c, nu/nu). The neoplastic cells were derived originally from an acinar cell carcinoma of the pancreas, induced in male Wistar rats by repeated injections of azaserine.

### *Animals*

Male nude mice (BALB/c, nu/nu) were obtained from Gibco, Animal Resource Laboratory, Madison, WI. Mice carrying or not $AT_3A$ and $AT_3B$ tumours were bled through the retro-orbital plexus, and the sera were stored at −20°C; tumours, pancreata, and livers were taken and processed for further analysis. Wistar rats of both sexes, weighing 160–180 g were obtained from Hilltop Laboratory Animals Inc., Scottdale, PA. Partial hepatectomy (Bucher, 1971) and partial pancreatectomy (Lehu & Fitzgerald, 1963) were performed on 160 g body weight male Wistar rats. The animals were housed in an air-conditioned room with 12 h light (7 a.m. to 7 p.m.) and dark cycle (7 p.m. to 7 a.m.). The animals were killed at 9 a.m. by bleeding through the abdominal aorta and serum was separated from blood and frozen at −20°C until analyzed; liver and pancreas of the normal, hepatectomized (24 h after) and pancreatectomized (72 h after) rats were resected, weighed and processed immediately for further analyses. The pancreata (pools of 25 per group) and the livers (pools of 3 per group) of foetal (20th day of gestation) and postnatal (4 days after birth) rats were processed similarly for analysis.

### *Serum lipid analyses*

Lipids were extracted from 0.5 to 1.0 ml of sera and analyzed by the methods previously described (Rao *et al.*, 1980*a*, 1983; 1984*a*, *b*). Neutral lipids were separated into cholesterol esters (CHE), free fatty acids (FFA), triglycerides (TG) and free cholesterol (FC) by thin-layer chromatography on silica gel G plates using the solvent system n-heptane:isopropyl ether:glacial acetic acid (60:40:2, v/v). The plates were air dried and the individual bands were identified by exposure to iodine vapour. The FC and CHE spots were scraped from the thin-layer chromatographic plates and eluted with 10 ml of chloroform and FC and CHE were estimated (Rao *et al.*, 1980*a*, *b*).

### *Lecithin: cholesterol acyltransferase (LCAT)*

LCAT activity was determined by the method of Wallentin and Vikrot (1980). The assay system consisted of 50 μl of serum, 20 μl of 0.01 M 5,5-dithiobis-2-nitrobenzoic acid, 30 μl of albumin stabilised 0.15 μCi of 7-(n)-[$^3$H] cholesterol (sp.act 5 Ci m mol$^{-1}$) dissolved in 0.1 M phosphate buffer, pH 7.4 in a final volume of 250 μl. The blank tubes received 20 μl of the above buffer and the test samples received 20 μl of 0.1M mercaptoethanol in phosphate buffer. At the end of the incubation at 37°C for 20 min, total lipids were extracted with 10 ml chloroform:methanol (2:1 v/v) and neutral lipids were separated by thin layer chromatography (Rao *et al.*, 1980*a*, *b*). The cholesterol and cholesterol ester spots were scraped and counted for tritium. LCAT activities were expressed as percent of cholesterol substrate converted to CHE ml$^{-1}$ serum h$^{-1}$ or nmol cholesterol esters formed ml$^{-1}$ serum h$^{-1}$.

### *Acyl CoA: Cholesterol Acyltransferase (ACAT)*

Tumours and tissues were homogenized at 4°C in 9 vol of 0.25 M sucrose with a Polytron (model PT10, Brinkman Instruments, Westbury, New York). Homogenates were centrifuged at 4000 rpm for 10 min and 100 μl of the supernatants were used for the assay of ACAT activity with 4-[$^{14}$C] cholesterol-albumin solution as the substrate (Heller, 1983). The assay system in a final volume of 0.7 ml contained 100 μl of the supernatant (0.5 to 1 mg of protein) 26.7 nM ATP, 1.07 mM coenzyme A and 0.15 μCi of [4-$^{14}$C]-cholesterol-albumin solution (sp act 55.7 mCi mmol$^{-1}$) in 0.2 ml of 0.1M phosphate buffer, pH 7.4. [4-$^{14}$C]-cholesterol solution was prepared by adding 2.25 μCi of [4-$^{14}$C] cholesterol in 30 μl of acetone to a solution of 75 mg of human serum albumin in 3 ml of 0.1 M phosphate buffer. Acetone was removed by evaporation under nitrogen. Incubations were carried out for 90 min at 37°C in a shaking water bath. At the end of the reaction, total lipids were extracted with 10 ml chloroform: methanol (2:1 v/v) and the neutral lipids were separated by thin layer chromatography (Rao *et al.*, 1980*a*, *b*). The cholesterol and cholesterol ester spots were scraped and counted for radiocarbon. Lipids were also extracted from 1 ml of the cytoplasmic extracts, and analyzed for cholesterol and cholesterol ester contents (Rao *et al.*, 1980*a*, *b*). Apparent ACAT activities were expressed as pmol of cholesterol esters formed $h^{-1}$ $mg^{-1}$ protein.

*Other procedures*

Proteins were estimated by the method of Lowry *et al.* (1951) using bovine serum albumin as the standard. Statistical analysis of the data was performed using analysis of variance (Steel & Torrie, 1980), and differences between means were considered significant if $P<0.05$.

*Reagents and chemicals*

4-$^{14}$C-cholesterol (sp act.55.7 mCi $mmol^{-1}$), [7 (n)-$^{3}$H] cholesterol (sp act. 5 Ci $mmol^{-1}$) were purchased from Amersham Corporation, Illinois. Human serum albumin, mercaptoethanol, and 5,5-dithiobis-2-nitrobenzoic acid were purchased from Sigma Chemical Company, St. Louis, MO. All other reagents and chemicals were of purity grade and were obtained from standard commercial sources.

**Table I** Serum cholesterol levels in nude mice and rats with proliferating tissues.

| *Sample* | *Group* | *Total cholesterol* | *Free cholesterol* | *Cholesterol ester* |
|---|---|---|---|---|
| Normal male nude mouse | a | 1.52† ±0.14 | 0.40 ±0.03 | 1.12 ±0.14 |
| $AT_3B$ host mouse | b | $0.96^{a}$ ±0.06 | 0.50 ±0.04 | $0.46^{a}$ ±0.03 |
| $AT_3A$ host mouse | c | $1.19^{b}$ ±0.05 | 0.51 ±0.07 | $0.68^{a,b}$ ±0.08 |
| Normal female rat | d | 1.0 ±0.10 | 0.17 ±0.02 | 0.81 ±0.11 |
| Postnatal mother rat | e | 0.91 ±0.04 | 0.22 ±0.02 | 0.69 ±0.05 |
| Pregnant rat | f | $1.30^{e}$‡ ±0.09 | 0.22 ±0.02 | $1.08^{e}$ ±0.08 |
| Normal male rat | g | 1.17 ±0.06 | 0.26 ±0.02 | 0.91 ±0.05 |
| Rat with regenerating pancreas | h | 0.98 ±0.09 | 0.22 ±0.01 | 0.76 ±0.08 |
| Rat with regenerating liver | i | $0.59^{g,h}$ ±0.03 | $0.16^{g,h}$ ±0.02 | $0.44^{g,h}$ ±0.03 |

†Values are in mg $ml^{-1}$ of sera. Each value is mean ± s.e. of 4 animals.

‡$P<0.05$ considered significant when compared with the group indicated.

**Results**

The serum cholesterol levels in nude mice and rats with proliferating tissues are presented in Table I. The total cholesterol levels in serum decreased significantly in host nude mice carrying slow growing $AT_3B$ but not fast growing $AT_3A$ tumours, whereas these levels were elevated in pregnant rat and lowered in rat with regenerating liver when compared with respective control groups. FC levels were unchanged in all groups except in rats with regenerating liver. During the active growth phase of the tumours (2 weeks after transplantation for $AT_3A$ and 4 weeks for $AT_3B$), the host nude mice showed a significant reduction in circulating CHE levels. At later stages of tumour growth, no such reduction in CHE levels in serum was seen (results not shown). The CHE levels increased significantly in the sera of pregnant rats near term when compared with postnatal mother rats 4 days after delivery, or with normal female rats. CHE levels decreased significantly in the rat serum after partial hepatectomy but not after partial pancreatectomy.

LCAT activity levels in the sera of host nude mice and in rats with proliferating tissues are presented in Table II. LCAT activity levels in the sera were calculated by subtracting the CHE counts of the blank tubes from the CHE counts of the test samples. From the total counts of the substrate added to the reaction tubes, the counts recovered in CHE spots were used to calculate the percent of CH converted to CHE $ml^{-1}$ serum. By estimating the FC content in each tube, nmol of CHE formed $h^{-1}$ $ml^{-1}$ serum were calculated. LCAT activity levels decreased significantly in host nude mice with $AT_3A$ and $AT_3B$ tumours, in pregnant rats and in rats with regenerating pancreata and regenerating liver, when compared with respective control groups.

The apparent ACAT activity levels in tumours and in tissues of nude mice and rats are presented in Table III. CHE counts of the blank tubes were subtracted from that of the CHE counts of the test samples. The CH and CHE contents in 100 μl of cytoplasmic extracts used in the assay were analyzed. The FC content present in each tube was estimated and from the amount of total counts of the substrate added in each tube, the pmol of CHE formed $h^{-1}$ $mg^{-1}$ protein were calculated. The apparent ACAT activity levels increased significantly in nude mouse tumours, foetal rat pancreata, postnatal rat liver, while these levels

**Table II** Lecithin:cholesterol acyltransferase activities in sera of nude mice and rats.

| Sample | Group | *Percent of CH converted to CHE* $ml^{-1}$ | *n moles CHE formed* $ml^{-1}, h^{-1}$ |
|---|---|---|---|
| Normal male nude mouse | a | 12.15† ±1.15 | 119.25 ±16.00 |
| $AT_3B$ host nude mouse | b | $5.37^{a}$‡ ±1.26 | $72.33^{a}$ ±6.05 |
| $AT_3A$ host nude mouse | c | $6.80^{a}$ ±0.54 | $83.75^{a}$ ±3.99 |
| Normal female rat | d | 14.50 ±1.24 | 73.97 ±5.02 |
| Postnatal mother rat | e | 10.82 ±1.23 | 60.23 ±4.89 |
| Pregnant rat | f | $6.07^{d,e}$ ±0.50 | $39.19^{d,e}$ ±1.62 |
| Normal male rat | g | 18.33 ±1.14 | 146.76 ±5.02 |
| Rat with regenerating pancreas | h | $10.9^{g}$ ±1.40 | $60.50^{g}$ ±12.53 |
| Rat with regenerating liver | i | $5.58^{g,h}$ ±0.50 | $22.50^{g,h}$ ±2.67 |

†Each value is Mean ± of s.e. of four animals.
‡$P<0.05$ considered significant when compared with the group indicated.
CH, cholesterol; CHE, cholesterol ester.

**Table III** The apparent Acyl-CoA: cholesterol acyltransferase activities in tumors and in tissues of nude mice and rats

| Sample | Group | *p mol CHE* $h^{-1}$ $mg^{-1}$ *protein* |
|---|---|---|
| Normal male nude mouse pancreas | a | 56.75† ±12.8 |
| $AT_3B$ tumour | b | $994.2^{a}$‡ ±279.4 |
| $AT_3A$ tumour | c | $1966.0^{a,b}$ +237.7 |
| Female rat pancreas | d | 47.48 ±43.2 |
| Postnatal rat pancreas | e | $535.3^{d}$ ±55.8 |
| Foetal rat pancreas | f | $1165.0^{d,e}$ ±191.11 |
| Female rat liver | g | 1893.0 ±234.74 |
| Postnatal rat liver | h | $7613.0^{g}$ ±607.7 |
| Foetal rat liver | i | $1275.0^{g,h}$ ±80.82 |
| Normal male rat liver | j | 5124.0 ±844.5 |
| Regenerating rat liver | k | $620.0^{j}$ ±41.39 |
| Normal male rat pancreas | l | 776.3 ±121.4 |
| Regenerating rat pancreas | m | 538.6 ±195.3 |

†Each value is mean ± s.e. of 4 adult animals or 4 pools consisting of 25 foetal or 25 postnatal pancreata; and 3 foetal or 3 postnatal livers.
‡$P<0.05$ considered significant when compared with the group indicated.
CHE, cholesterol ester.

decreased significantly in foetal and regenerating rat liver and remained unchanged in regenerating pancreas when compared with respective control tissues.

### Discussion

Previous studies showed enhanced cholesterogenesis in tissues with active proliferating cells (Rao *et al.*, 1983). While normal tissues showed strictly regulated cholesterol synthesis, as evidenced by a drop in cholesterogenesis when cell proliferation ceased, cholesterol synthesis appeared to be deregulated in tumour cells (Rao *et al.*, 1984*a*, Siperstein, 1984). Both fast and slow growing pancreatic tumours *in vivo* showed loss of feedback control of cholesterol synthesis and their growth rates were found to be related at least in part to their rates of cholesterol synthesis (Rao *et al.*, 1983; 1984*a*). In vitro, the same tumour cells showed feedback control of cholesterol biosynthesis. These results suggested that *in vivo* the tumour cell down regulated LDL receptors while expressing these receptors *in vitro* (Rao *et al.*, 1984*a*). Thus it appears that the influx of circulating CHE is reduced into the tumour cell not only by down regulation of receptor mediated endocytosis of serum CHE but also by a significant reduction in serum CHE levels during active growth phase of $AT_3A$ and $AT_3B$ tumours (Table I). It was shown that in rats carrying Morris minimal deviation hepatoma, during the early stages of tumour growth, total neutral lipids and CHE decreased while FC levels increased, and at later stages, there was hyperlipidaemia (Wood *et al.*, 1982). However, when host rats carrying Yoshida ascites hepatoma AH-130 were fasted overnight, no changes were seen in serum lipids (Ruggieri & Fallani, 1979). Serum cholesterol levels decreased after partial hepatectomy but not after partial pancreatectomy. It is known that 24 h after partial hepatectomy serum total cholesterol levels, total lipids, and HDL decrease, resulting in fatty liver and accumulation of cholesterol esters in liver (Narayan *et al.*, 1968; Fex & Wallinder, 1973). As in previously reported studies (Khamsi *et al.*, 1972; Argiles & Herrera, 1981), in the present study total cholesterol and CHE levels increased in serum of pregnant rat near term. Even though the mother shows high circulating cholesterol levels, very little cholesterol passes from mother to the foetus (Calandra *et al.*, 1975). The foetus has a lower plasma lipid concentration (Argilis & Herrera, 1981) and maintains enhanced *de novo* cholesterogenesis (Rao *et al.*, 1983) compared to the mother. These results suggest that during cell proliferation *de novo* cholesterogenesis is enhanced (Rao *et al.*, 1983, 1984*a*; Pani *et al.*, 1984) and circulating CHE levels are reduced, resulting probably in less influx of serum cholesterol.

LCAT activities in the sera of host nude mice with $AT_3A$ and $AT_3B$ tumours and in rats with proliferating tissues decreased significantly (Table II). This change coincides with cell proliferation and enhanced *de novo* cholesterogenesis (Rao *et al.*, 1983, 1984*a*; Pani *et al.*, 1984). Thus pregnant rats and rats with regenerating pancreas and liver had significantly lower LCAT activities than their normal counterparts. One day after partial hepatectomy LCAT activities were shown to fall rapidly and were later restored after liver regeneration (Fex & Wallinder, 1973). Similarly LCAT activities decreased in sera of rats after lead induced liver hyperplasia. During peak DNA and cholesterol biosynthesis LCAT activities decreased significantly and later restored to normal levels with decreases in DNA and cholesterol synthesis (Pani *et al.*, 1984). While data concerning LCAT activity in foetus is not available, in cord blood and in the newborn rat it is significantly lower than the adult rats (Lacko *et al.*, 1972). LCAT activity, as in the present study, was found to be higher in males than in females (Soler-Argilaga *et al.*, 1977). No data concerning the levels of LCAT activities in host animal sera carrying tumours were reported in the literature.

A decrease in LCAT activity during cell proliferation and enhanced cholesterol synthesis is in accordance with proposed role of LCAT in sterol efflux, esterification and influx (Fielding & Fielding, 1982). LCAT activity in serum of mice and rats with proliferating tissue was lowered (Table II) so that the CHE levels decreased resulting in less efflux of free CH from the cells and less influx of CHE into the cells. Such a reduced influx will decrease the formation of FC from internalized CHE and remove the inhibition of FC on HMG-CoA reductase, resulting in enhanced cholesterogenesis. The pregnant rat has low LCAT activities and high CHE content in the serum. The reason for this low LCAT activity is not known. Probably the low LCAT activities may result from reduced synthesis of LCAT by liver or by less availability of apo-A1, the cofactor for LCAT activity or inhibition by apo A-II (Frohlich *et al.*, 1982).

Cells also possess the ability to esterify *de novo* synthesized FC by the action of ACAT. Both HMG-CoA reductase and ACAT are located in endoplasmic reticulum (Bucher *et al.*, 1960; Goodman *et al.*, 1964). The regulation of ACAT activity is not known, but recent evidence indicates that intracellular cholesterol serves both as a substrate and non-substrate modulator (Hashimoto *et al.*, 1983). In the present study, apparent ACAT activity (Table III) increased in nude mouse tumours and in foetal pancreata. In the case of foetal liver near term, ACAT activity decreased when compared to adult liver suggesting that *de novo* cholesterogenesis was at a declining stage on the twentieth day of gestation when compared to earlier stages of the foetus. The apparent ACAT activity in the newborn rat liver was high because of the influence of suckling the mother's milk and the free cholesterol absorbed through the gut probably was converted to cholesterol esters. It is not surprising to find low apparent ACAT activities in liver at 24 h after partial hepatectomy (PH) as it was shown that HMG-CoA reductase activity peaks at 8 h after PH, *de novo* cholesterogenesis at 16 h after PH and DNA synthesis at 22–24 h after PH (Trentalance *et al.*, 1984). Similarly, the apparent ACAT activities were unchanged in pancreas 72 h after partial pancreatectomy, since peak DNA synthesis was shown to occur at this stage (Lehu & Fitzgerald, 1963) and as in liver (Bucher, 1971), HMG-CoA reductase activity, and *de novo* cholesterogenesis may peak earlier than DNA synthesis (Trentalance *et al.*, 1984).

Taken together, the results of the present study indicate that in addition to stimulation of HMP pathway of glucose metabolism (Rao *et al.*, 1984*a*), LCAT, ACAT and serum CHE levels are regulated during enhanced *de novo* cholesterogenesis. The exact functional role of this coordinated regulation between cholesterol esterification and enhanced cholesterogenesis in the modulation of cell proliferation needs to be established by further experimentation.

We thank Drs. B. Lombardi and D.H. Van Thiel for helpful discussions. These studies were supported by National Dairy Council, Samuel Emma Winters Foundation (KNR) and by CNR #84.00608.44, Rome, Italy, (PP)..

### References

ANDERSON, J.M. & DIETSCHY, J.M. (1977). Regulation of sterol synthesis in 15 tissues of rat: II Role of rat and human high and low density plasma lipoproteins and of rat chylomicron remnants. *J. Biol. Chem.*, **252**, 3652.

ARGILES, J. & HERRERA, E. (1981). Lipids and lipoproteins in maternal and fetus plasma in the rat. *Biol. Neonate.*, **39**, 37.

BUCHER, N.L.R. (1971). Experimental aspects of hepatic regeneration. *New Engl. J. Med.*, **277**, 686.

CALANDRA, S., QUARTAROLI, G.C. & MONTAGUTI, M. (1975). Effect of cholesterol feeding on cholesterol biosynthesis in maternal and fetal rat liver. *Europ. J. Clin. Invest.*, **5**, 27.

COLEMAN, P.S. & LAVIETES, B.B. (1981). Membrane cholesterol and tumorigenesis. *CRC Critical Rev. Biochem.*, **11**, 341.

DREVON, C.A., ATTIE, A.D., PANGBURN, S.H. & STEINBERG, S. (1981). Metabolism of homologous and heterologous lipoproteins by cultured rat and human skin fibroblasts. *J. Lipid. Res.*, **22**, 37.

FEX, G. & WALLINDER, L. (1973). Liver and plasma cholesterol ester metabolism after partial hepatectomy in the rat. *Biochim. Biophys. Acta.*, **316**, 91.

FIELDING, C.J. & FIELDING, P.E. (1982). Cholesterol transport between cells and body fluids. *Med. Clin. N. America.*, **66**, 363.

FROHLICH, J., McLEOD, R. & HON, K. (1982). Lecithin: cholesterol acyltransferase (LCAT). *Clin. Biochem.*, **15**, 269.

HASHIMOTO, S., DREVON, C.A., WEINSTEIN, D.B., BERNETT, J.S., DAYTON, S. & STEINBERG, D. (1983). Acitivity of Acyl-CoA: cholesterol acyltransferase and 3-hydroxy-3-methylglutaryl-CoA reductase in subfractions of hepatic microsomes enriched with cholesterol. *Biochim. Biophys. Acta.*, **754**, 126.

HELLER, F.R. (1983). Cholesterol esterifying capacity of various organs in cholesterol fed guinea pigs. *Lipids*, **18**, 18.

KHAMSI, F., MERAKATZ, J. & SOLOMON, S. (1972). The conversion of acetate to cholesterol in the fetus of the baboon and the transfer of cholesterol from mother to fetus. *Endocrinology*, **91**, 6.

LACKO, A.G., RUTENBERG, H.L. & SOLOFF, L.A. (1972). On the rate of cholesterol esterification cord blood serum. *Lipids*, **7**, 426.

LEHU, M. & FITZGERALD, P.J. (1963). Pancreatic acinar cell regeneration. *Am. J. Pathol.*, **53**, 513.

LOWRY, O.H., ROSEBROUGH, J.J., FARR, A.L. & RANDALL, R.J. (1951). Protein measurement with Folin phenol reagent. *J. Biol. Chem.*, **193**, 262.

NARAYAN, K.A., ELIZABETH, S., MARY, G.E.S. & KUMMEROW, F.A. (1968). Rat serum lipoproteins after partial hepatectomy. *Proc. Soc. Exp. Biol. Med.*, **129**, 6.

PANI, P., DESSI, S., RAO, K.N., BATETTA, B. & LACONI, E. (1984). Cholesterol synthesis in lead induced liver hyperplasia in Wistar rats. *Toxicol. Pathol.*, **12**, 162.

RAO, K.N., KATYAL, S.L., IAMMARINO, R.M. & LOMBARDI, B. (1980*a*). Acute hemorrhagic pancreatic necrosis, in mice: alterations in pancreatic lipase activity, pancreas lipids, and serum lipoproteins. *Digestion*, **20**, 314.

RAO, K.N., KOTTAPALLY, S. & SHINOZUKA, H. (1983). Lipid composition and HMG-CoA reductase activity of acinar cell carcinoma of rat pancreas. *Biochim. Biophys. Acta.*, **459**, 74.

RAO, K.N., KOTTAPALLY, S. & SHINOZUKA, H. (1984*a*). Acinar cell carcinoma of rat pancreas: Mechanism of deregulation of cholesterol metabolism. *Toxicol. Pathol.*, **12**, 62.

RAO, K.N., MISRA, D.N., KELLY, R.H. & SHINOZUKA, H. (1980*b*). Alterations in glycoproteins and lipids in azaserine-induced acinar cell carcinoma of rat pancreas. *Cancer Lett.*, **10**, 19.

RAO, K.N., SHINOZUKA, H., KUNZ, H.W. & GILL III, T.J. (1984*b*). Enhanced susceptibility to a chemical carcinogen in rats carrying MHC-linked genes influencing development (grc). *Int. J. Cancer.*, **34**, 113.

RAO, K.N., TAKAHASHI, S. & SHINOZUKA, H. (1980*c*). Acinar cell carcinoma of rat pancreas. Growth in cell culture and in nude mice. *Cancer. Res.*, **40**, 592.

RUGGIERI, S. & FALLANI, A. (1979). Lipid composition of Yoshida ascites hepatoma and of livers and blood plasma from host and normal rats. *Lipids*, **14**, 323.

SIPERSTEIN, M.D. (1984). Role of cholesterogenesis and isoprenoid synthesis in DNA replication and cell growth. *J. Lipid. Res.*, **25**, 1462.

SOLER-ARGILAGA, C., RUSSEL, R.L., GOH, E.H. & HEIMBERG, H. (1977). Hepatic secretion and turnover of serum phosphatidylcholine cholesterol acyltransferase in male and female rats. *Biochim. Biophys. Acta.*, **488**, 69.

STEEL, R.G.D. & TORRIE, J.H. (1980). *Principles and procedures of statistics: a biomedical approach* p. 137, 2nd Ed. McGraw-Hill, New York.

TRENTALANCE, A., LEONI, S., MANGIANTINI, M.T. & 6 others. (1984). Regulation of 3-hydroxy-3-methylglutaryl-coenzyme A reductase and cholesterol synthesis and esterification during the first cell cycle of liver regeneration. *Biochim. Biophys. Acta.*, **794**, 142.

WALLENTIN, L. & VIKROT, O. (1975). Evaluation of and *invitro* assay of lecithin: cholesterol acyltransferase of human plasma. *Scand. J. Clin. Lab. Invest.*, **35**, 661.

WOOD, R., ZOELLER, A. & MATOCHA, M. (1982). Effect of hepatoma on host liver, heart and liver lipids as tumor growth progresses. *Lipids.*, **17**, 771.